# The broiler chicken as a signal of a human reconfigured biosphere

**DOI:** 10.1098/rsos.180325

**Published:** 2018-12-12

**Authors:** Carys E. Bennett, Richard Thomas, Mark Williams, Jan Zalasiewicz, Matt Edgeworth, Holly Miller, Ben Coles, Alison Foster, Emily J. Burton, Upenyu Marume

**Affiliations:** 1 School of Geography, Geology and the Environment, University of Leicester, Leicester LE1 7RH, UK; 2 School of Archaeology and Ancient History, University of Leicester, Leicester LE1 7RH, UK; 3 Department of Classics and Archaeology, University Park, University of Nottingham, Nottingham NG7 2RD, UK; 4 School of Animal, Rural and Environmental Sciences, Nottingham Trent University, Nottingham NG25 0QF, UK; 5 School of Agriculture Science, North West University, P Bag X 2046, Mmabatho 2735, South Africa

**Keywords:** biosphere, chicken, Anthropocene, agriculture, evolution

## Abstract

Changing patterns of human resource use and food consumption have profoundly impacted the Earth's biosphere. Until now, no individual taxa have been suggested as distinct and characteristic new morphospecies representing this change. Here we show that the domestic broiler chicken is one such potential marker. Human-directed changes in breeding, diet and farming practices demonstrate at least a doubling in body size from the late medieval period to the present in domesticated chickens, and an up to fivefold increase in body mass since the mid-twentieth century. Moreover, the skeletal morphology, pathology, bone geochemistry and genetics of modern broilers are demonstrably different to those of their ancestors. Physical and numerical changes to chickens in the second half of the twentieth century, i.e. during the putative Anthropocene Epoch, have been the most dramatic, with large increases in individual bird growth rate and population sizes. Broiler chickens, now unable to survive without human intervention, have a combined mass exceeding that of all other birds on Earth; this novel morphotype symbolizes the unprecedented human reconfiguration of the Earth's biosphere.

## 1. Background

**Figure 1.**
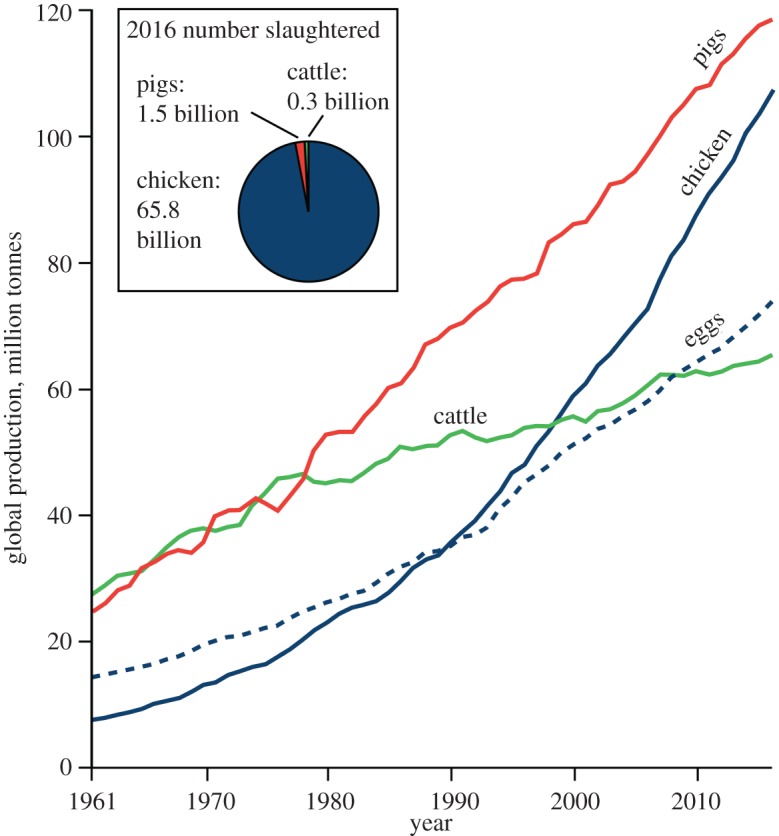
Global consumption of domesticated chicken, pig and cattle from 1961 to 2016. Chicken is the most common meat consumed, with a standing population in 2016 of over 22.7 billion. The number of chickens slaughtered per year is an order of magnitude larger than that of pigs or cattle. Data compiled from UN Food and Agriculture Organization, FAOSTAT, at faostat.fao.org, accessed November 2018.

Accelerating human-driven physical, chemical and biological changes to the Earth system have been profound, sparking suggestions of a new geological epoch, the Anthropocene [1]. Human consumption trends have driven unprecedented changes to the Earth's biosphere [2–4]. Populations of wild animal groups have plummeted in recent decades [5], while human and livestock populations have risen [6]. The biomass of humans and their domesticated animals (including livestock) now outweighs that of all wild terrestrial vertebrates [7,8]. Understanding the nature of this change is important to help protect biodiversity. Domesticated chickens (*Gallus gallus domesticus* Linnaeus, 1758) are a striking example of a human reconfigured biosphere. They are the world's most numerous bird with a standing population of 22.7 billion [6]. This population is an order of magnitude greater than the standing stocks of the most abundant wild bird species (red-billed quelea approximately 1.5 billion, house sparrow approximately 0.5 billion, rock dove approximately 0.25 billion [9]), other farmed birds (turkeys approximately 0.3 billion, geese approximately 0.3 billion, ducks approximately 1.1 billion [6]) and farmed pigs and cattle (figure 1). In Europe, the population of domesticated chickens in 2009 (1.9 billion) was greater than the combined population of the 144 most numerous wild bird species (1.6 billion) [10]. It is likely to be the largest standing population of a single bird species in Earth's history.

Chicken-meat consumption is growing faster than any other meat type and is soon to outpace pork (figure 1). Expanding consumption in developing countries is driving the trend [11]. This has had a profound impact on the biology of the broiler (meat-chicken). Since the Chicken-of-Tomorrow Program in the early 1950s, launched to encourage the development of higher meat-yielding birds [12], chickens have undergone extraordinary changes. From the mid-twentieth century to the present, broiler growth rates have soared, with up to a fivefold increase in individual biomass [13,14]. Here, we document the biology of the broiler from the Roman era to the present and discuss whether the biological changes to the broiler are distinctive enough to make them a marker species of the proposed Anthropocene Epoch. Changes to the broiler skeleton [15], diet and genetics [16] (preserved as geochemical signatures in their bones) have the potential to be fossilized.

**Figure 2.**
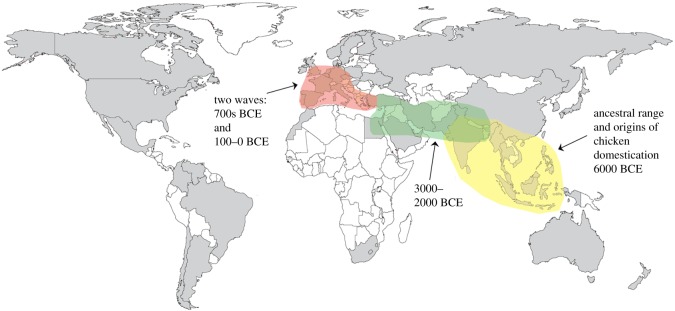
Industrial broiler production and archaeological record of domestic chickens in the Old World. Colour key: grey: countries with industrial broiler production (vertical integration systems); white: countries with no industrial production, but chicken meat is consumed. Countries with vertical integration systems have been reported in the literature, or they produce large volumes of chicken meat (greater than 500 000 tonnes yr^−1^ from 2013 to 2014 [6]).

The current dominance of *Gallus gallus domesticus* as the world's most numerous bird species would not be possible without modern technology. The system of industrial chicken production and its export around the world has facilitated surging chicken-meat consumption. Separate broiler breeding units, farms, slaughterhouses, processing plants and marketing are coordinated into one system called vertical integration. First implemented in the southern USA in the 1950s, vertical integration systems now account for 97% of USA broiler production [17]. Industrial chicken farming is now widespread around the globe (figure 2) and has enabled the rapid rise in broiler production from the 1950s onwards (figure 1; electronic supplementary material, figure S1). In 2006 it was estimated that 70% of broilers were intensively reared [18]. The system epitomizes efficient and high volume production from a human perspective: in the USA the top broiler production firm, Tyson Foods, slaughtered 35 million chickens per average week in 2012 [17]. Retailers of chicken are some of the most successful and globalized, for example KFC, the world's largest chicken meat retailer, has over 25 500 stores in 125 countries worldwide (www.kfc.com, accessed February 2018).

The domestic chicken originated from the red jungle fowl (*Gallus gallus* Linnaeus, 1758), native to tropical S/SE Asia [19]. Contemporary, non-indigenous red jungle fowl occur in the Americas, Australia, Europe and Africa, due to deliberate human translocation, and are adapted to a much wider climatic range than indigenous birds [19]. The fossil record of the red jungle fowl is poorly known, but molecular-clock estimates place the origin of the order Galliformes (pheasants, jungle fowl and relatives) in the early Cretaceous [20]. However, the archaeological record of chicken domestication and husbandry is relatively well documented (figure 2). Archaeological studies had suggested that chickens were domesticated *ca* 8000 years ago, although ongoing DNA and dating research is likely to bring this date forward [21]. Domesticated chicken bones are recorded from the Indus valley as early as 2500–2100 BCE [22]. The time-transgressive spread of domesticated chickens out of Asia is contemporaneous with the establishment of new trade routes [23]. For example, chickens were introduced to the Iberian Peninsula by Phoenician traders in the first century CE and Spanish colonists introduced domesticated chickens to the New World in 1500 CE [23].

## 2. Methods

Since the early 1990s the Museum of London Archaeology (formally the Museum of London Specialist Services and Museum of London Archaeological Service) has systematically recorded zooarchaeological data from developer-funded excavations in London. Included among this dataset is an enormous archive of animal bone measurements taken using the standard set out by von den Driesch (1976) [24]. In this study, 486 individual tibiotarsus distal breadth measurements from 74 sites from the city were analysed and compared with modern broiler data. The distal width was measured in preference to length of the tibiotarsus because the majority of bone specimens were broken. To avoid potential confusion with age-related size change, measurements from archaeological specimens were all derived from skeletally mature birds. To facilitate the identification of temporal trends and accommodate the majority of the archaeological data, bone measurements were placed into 12 overlapping temporal groups (electronic supplementary material, table S1). Assemblages that were broadly or insecurely dated assemblages (i.e. bones from contexts with residual pottery) were excluded from analysis. It is possible that the group of domestic fowl bones include guinea fowl (*Numida meleagris* L., 1758) and pheasant (*Phasianus colchicus* L., 1758) bones, which are morphologically similar to chicken [25,26]. This is pertinent to the archaeological record of early chicken domestication: the re-examination of early Holocene bones from northern China reputed to provide early evidence of chicken domestication, revealed that most of the bones were from pheasants rather than chickens [27]. However, pheasant and guinea fowl are only identified rarely so it is assumed that most fowl bones derive from chicken. A non-parametric Mann–Whitney pairwise comparison was run using the statistical software PAST [28] to examine the statistical significance of broiler skeletal size (electronic supplementary material, table S1). The datasets supporting this article are included in the electronic supplementary material.

Data on broiler and other livestock populations was sourced from the FAO (Food and Agriculture Organization of the United Nations) website (http://faostat3.fao.org) and the United States Department of Agriculture statistics service (www.nass.usda.gov). Data on chicken growth rates, osteo-pathologies and bone collagen isotopes were synthesized from numerous publications (cited in the figure captions).

## 3. Results

**Figure 3.**
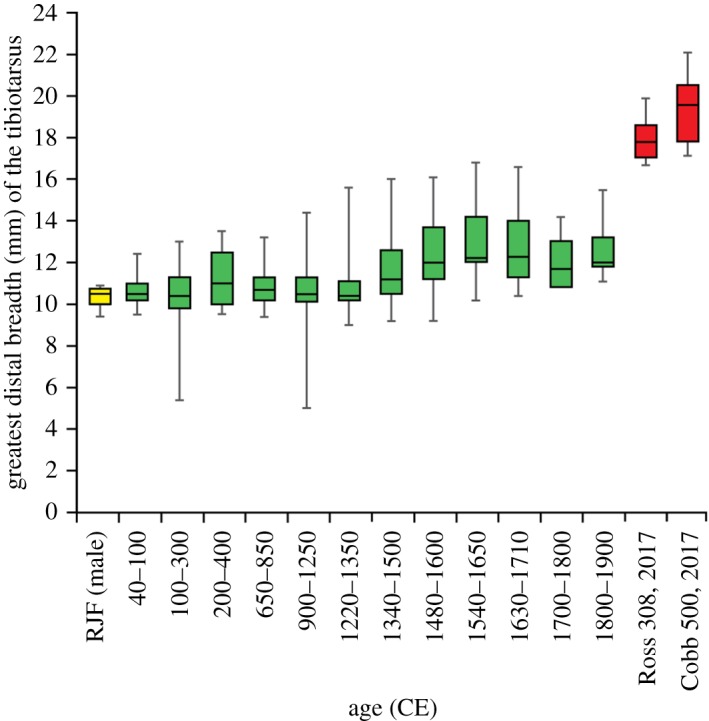
Box and whisker plot of domestic chicken bone size through time. Measurements are of the width of the tibia (greatest distal breadth, Bd, in mm), *N* = 522. Archaeological data derived from sites throughout London from 1220 CE to 1900 CE [29] augmented with new measurements from the Roman and early medieval periods, compared to measurements of a cross-bred red jungle fowl (RJF) [30] and six-week old modern broilers from South Africa (Cobb 500 breed, sex unknown) [31] and England (Ross 308 breed, males). A statistical analysis of the dataset is given in electronic supplementary material, table S1.

**Figure 4.**
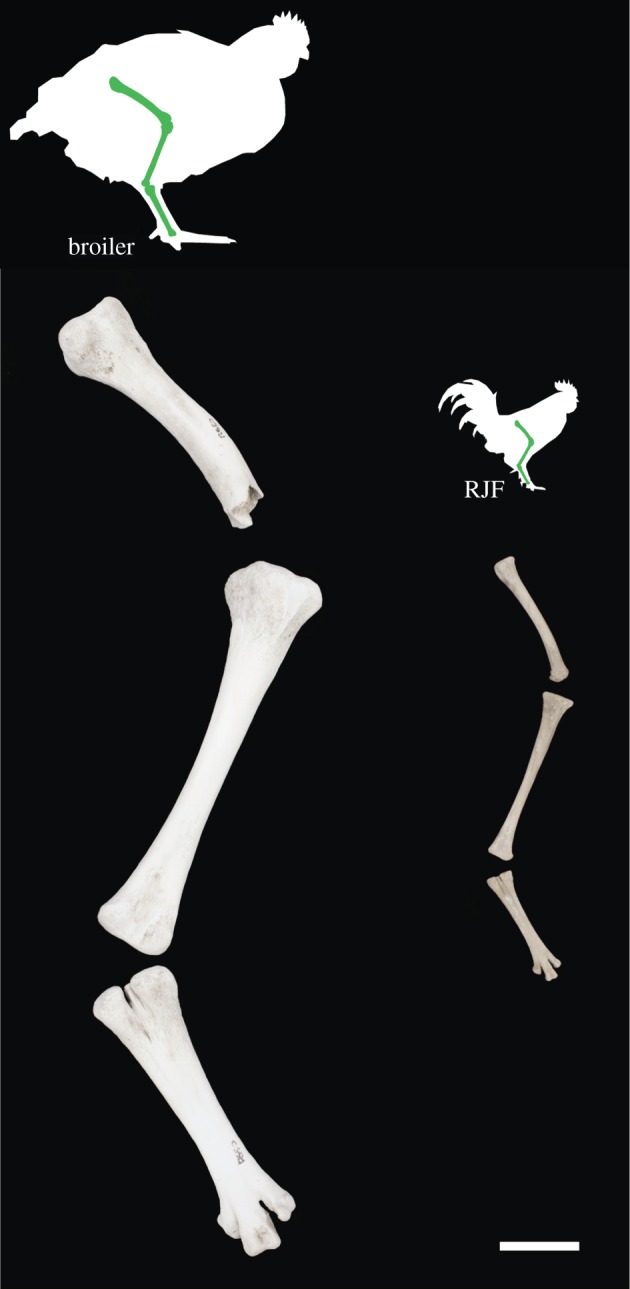
Limb bones (femur, tibiotarsus, tarsometatarsus) of a modern broiler and a red jungle fowl. A juvenile broiler and red jungle fowl silhouette with limb bones are shown at proportionate size to each other for comparison. Broiler limb bones (left): male, five weeks of age and a Cobb, specimen R650, from the School of Archaeology and Ancient History, University of Leicester. Cross-bred red jungle fowl limb bones (right): male, six weeks of age and of modern breeding stock from an ancestral line, specimen NHMUK S/2009.1.11, from the Ashdown Collection, Natural History Museum, Tring. At slaughter age the over-sized but immature skeleton of the broiler is characteristically poorly ossified and relatively featureless. Image copyright of the Trustees of the Natural History Museum, London. Scale bar 20 mm.

The size of chicken bones from multiple archaeological sites in London, UK, is recorded from the Roman era to the end of the nineteenth century (figure 3) compared against a red jungle fowl and two broiler datasets. Modern broiler skeletons are significantly larger than both the wild progenitor bird and archaeological domestic chickens (figure 3; electronic supplementary material, table S1). The greatest distal breadth of the tibiotarsus (which provides a proxy of body mass) is in some specimens twice the width in a six-week-old modern broiler than in an adult red jungle fowl (figure 3). More dramatic, is a direct comparison of juvenile broiler and red jungle fowl lower limb bones of the same age, which shows a tripling in width and a doubling in length (figure 4). From the Roman era until 1340 the distal widths of domesticated chicken tibiotarsi in London were similar to those of their red jungle fowl ancestor (electronic supplementary material, table S1). Sustained increases in the size of chickens from 1340 to 1650 (figure 3) are concurrent with size increases in other domesticated livestock [29]. The average distal tibiotarsus width measurements from 1650 up until 1900 remain fairly constant (figure 3).

**Figure 5.**
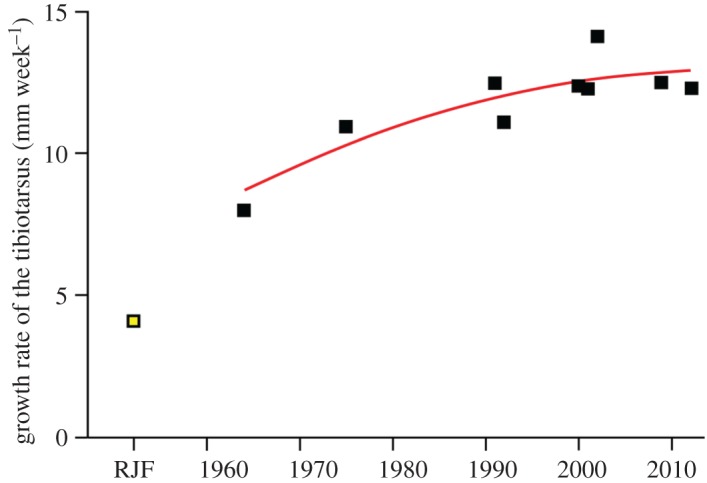
Accelerating growth rate of broilers throughout the second half of the twentieth century. The tibiotarsus bone is used as a proxy for skeleton growth, *N* = 10. Records of tibiotarsus growth rates were obtained from broilers reared in 1964: New Hampshire/Barred Rock cross-breed, USA [32]; 1975: unspecified commercial broilers, Canada [33]; 1991: Vantress/Arbor Acre cross-breed, USA, males [34]; 1992: Cobb, Nigeria [35]; 2000: Ross, Brazil [36]; 2001: unspecified commercial broilers, Turkey [37]; 2002: Ross/Arbor Acre cross-breed, USA [38]; 2009: unspecified commercial broilers, USA [39]; 2012: Ross, Poland [40]. These are compared to the growth rate of the cross-bred red jungle fowl, UK [30]. A smoothing spline with a smoothing factor of 4.35 [41] fitted to the 1964 to 2012 data indicates an increase in the growth rate of broilers from 1964 to present.

While the archaeological record for chickens indicates alterations in bone morphology related to domestication, the speed and scale of changes escalated considerably in the second half of the twentieth century. A surge in chicken production from the 1950s onwards (figure 1) has resulted in an increase over this period in the mass of individual birds (electronic supplementary material, figure S1). We quantify how the growth rate of broilers (from juvenile to adult) has changed over the twentieth century by a compilation of data from several commercial breeds (figure 5; electronic supplementary material, figure S2). There has been a steady increase in growth rate since 1964 and the growth rate of modern broilers is now three times higher than that of the red jungle fowl. However, data from the twenty-first century show that the growth rate is slowing (figure 5; electronic supplementary material, figure S1) and may be reaching a plateau.

**Figure 6.**
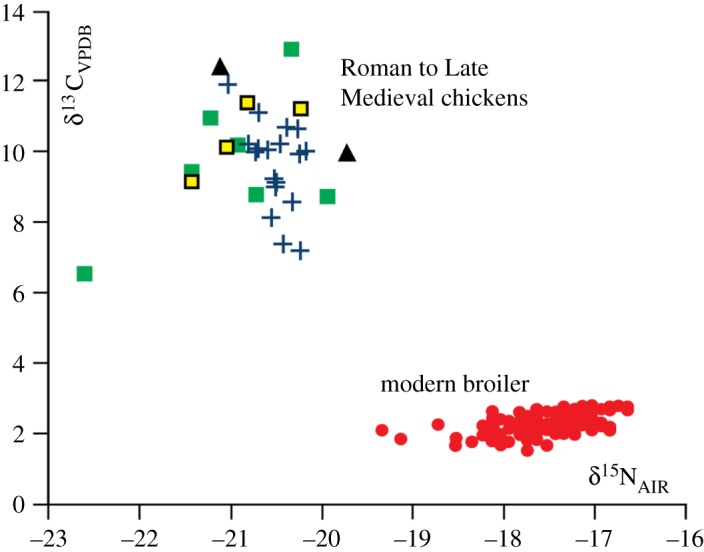
Carbon (δ^13^C) and nitrogen (δ^15^N) isotope values of chicken bone collagen. Symbols relate to chicken bone collagen analysed from different periods: green squares: Roman (*N* = 7); black triangles: Anglo-Saxon (*N* = 2); yellow squares: High Medieval (*N* = 4); blue crosses: Late Medieval (*N* = 18); red circles: modern (*N* = 161). The data illustrate a change in diet towards a greater contribution of C4 cereal grains, predominantly maize, since the 1950s, which has altered the isotope values in broiler chicken bone collagen. Data compiled from various published sources [42–44]. The results of a discriminant analysis show that the modern broiler isotope data is significantly different from the archaeological isotope data (electronic supplementary material, figure S3).

A change in the diet of domesticated chickens to produce high meat-yields is detected in the analysis of dietary carbon (δ^13^C) and nitrogen (δ^15^C) isotopes (figure 6). Chicken bone collagen isotopes from the Roman, Anglo-Saxon, High Medieval and Late Medieval periods are distinct from those of modern broilers (figure 6; electronic supplementary material, figure S3).

Multiple and significant osteo-pathologies arise as a consequence of the accelerated growth rate of broilers (electronic supplementary material, figure S4). These pathologies are common in broilers, but are only rarely observed in archaeological bones: for example, tibial dyschondroplasia (poor ossification of the tibia leading to lameness) is recorded in a turkey from nineteenth-century London [45], but has never been reported in chicken bones from archaeological contexts.

## 4. Discussion

The intensive production of broilers and rising rates of consumption (figure 1) increases the standing biomass of domesticated chickens year on year. Over 65.8 billion meat-chicken carcasses were consumed globally in 2016 [6] and this is set to continue rising. This may be an underestimate, given the standing population of 22.7 billion and lifespan of five to seven weeks. The FAO states that this number is aggregated from statistics that may be official, semi-official, estimates, or calculated data. The lifespan of broiler chickens is short (five to seven weeks) compared to egg-laying hens (1 year), both of which are slaughtered at a young age for economic reasons. The contrast between the lifespan of the ancestral red jungle fowl (3 years [46] to 11 years (Tommaso Pizzari 2018, personal communication) in captivity) and that of broilers means that the potential rate of carcass accumulation of chickens is unprecedented in the natural world. Irrespective of the number of broiler chickens killed per annum, the standing population of 22.7 billion chickens is striking and mirrors other population data comparing domesticated with wild animal populations; for example, the biomass of domesticated cattle is 250 times higher than that of elephants [8]. The standing biomass of domesticated poultry, mostly chickens, has been calculated as 5 Mt C, about three times higher than the total biomass of all wild bird species combined [7]. The rise in the number of domesticated chickens over recent decades mirrors the decline in the population numbers of wild bird species, particularly those that are the most common [5,10]. This mono-specific vast bird biomass is unprecedented in Earth's recent history and perhaps also in Earth's geological history. While fossil bird populations are difficult to estimate, it is thought that the most common wild bird in human history, the passenger pigeon, had a population of 3–5 billion in the 1800s [47].

The global range of modern broilers, in comparison with the geographically restricted range of their jungle fowl ancestors, is in part a factor of their climate-controlled (temperature, humidity and light) housing conditions [48]. The vertical integration farming system of industrial production is reliant on the technosphere (the global emergent system that includes humans, technological artefacts, and associated social and technological networks) [49]. Broiler farming is undertaken within a complex mechanized system that operates with the integration of computer software, electricity, transportation vehicles, refrigeration, feed processing factories and more [17]. This is epitomized in the life cycle of intensively farmed broilers: eggs are laid in broiler breeder facilities and transported to hatcheries, where eggs are incubated artificially for 21 days [50]. After hatching, the 1-day-old chicks are transported to high-capacity finishing units housing up to 50 000 individuals in climate-controlled sheds [51]. For the first week of life, chicks are kept at temperatures of 32°C to 35°C and relative humidity of 60% to 70% [48]. At five to seven weeks old, broiler chickens are transported to the slaughterhouse, where most waste products (feathers, manure, blood etc.) are recycled via anaerobic digestion, incineration and rendering into edible by-products [52], all technology-dependent.

Breeding by natural selection has been modified by human-directed selection. While the size of the domesticated chicken in historical times was little different to the red jungle fowl (figure 3), domestic chicken bone morphology shows that selective breeding practices took place as early as the sixteenth century [53,54]. Chickens from the late twentieth century are markedly different in terms of size (figures 3 and 4), growth rate (figure 5) and body shape. The change in body mass and body shape has been visually documented by photographs of broiler breeds throughout ontogeny from 1957, 1978 and 2005 [14]. Broilers from a 1957 breed are between one-fourth and one-fifth of the body weight of broilers from a twenty-first century breed [13,14]. The modern broiler is a distinctive new morphotype with a relatively wide body shape, a low centre of gravity [13] and multiple osteo-pathologies. If left to live to maturity, broilers are unlikely to survive. In one study, increasing their slaughter age from five weeks to nine weeks resulted in a sevenfold increase in mortality rate [55]: the rapid growth of leg and breast muscle tissue leads to a relative decrease in the size of other organs such as the heart and lungs, which restricts their function and thus longevity [56]. Changes in the centre of gravity of the body, reduced pelvic limb muscle mass and increased pectoral muscle mass cause poor locomotion and frequent lameness [15]. Unlike most other neobiota, this new broiler morphotype is shaped by, and unable to live without, intensive human intervention.

Naturally omnivorous, the diet of the broiler chicken has become more grain-based with approximately 60% of broiler feed composed of cereals such as maize, wheat and barley [18]. Diets vary globally, with maize most commonly used in the USA because it has a higher nutrient value than other cereals [57]. Additions to the dietary cereals can include fishmeal [18] and re-processed hatchery and broiler waste (egg shells, chicks and chickens) [13]. The alteration to chicken diets is designed to reduce the amount of feed used while increasing meat yields [13]; however, it also homogenizes nutritional intake, eliminating a source of natural variation within the stock (figure 6). The current plateau in broiler growth rates may not be maintained, with current research focusing on new technologies to increase the protein intake of broiler diets by using insect meal instead of plant proteins [58].

The genetic make-up of the modern broiler morphospecies is equally striking. The domestic chicken's genetic make-up differs from the ancestral red jungle fowl, in terms of deletions and mutations, some of which relate to the modification of the animal for maximum growth [59]. For example, the thyroid stimulating hormone receptor (TSHR) allele has a pivotal role in metabolic regulation and photoperiod control of reproduction, allowing domestic breeds to reproduce year-round [59]. This genetic marker could be used to recognize *Gallus gallus domesticus* in the future fossil record, in the way that the β-carotene dioxygenase 2 gene associated with yellow skin colour resulting from a diet of maize may be identified in archaeological chicken bones from approximately 500 years ago [60]. Three companies worldwide supply 90% of broiler chicks and selective breeding has resulted in 50% or more of genetic diversity loss in commercial lines compared with ancestral breeds [16].

Global human trends towards increased animal protein consumption, along with an increased human population, is impacting on land use and wild species populations [5]. Chickens have the greatest feed efficiency of any farmed animal species [61], but their numerical dominance is reflected in feed consumption. In the USA the combined feed consumption by broiler and egg-laying chickens has been calculated as 58 Mt (×10^9^ kg) of dry concentrates (grains and by-products) per year; the greatest feed volume of any farmed animal group [62]. Overall, the land area required to produce feed for chickens is lower than for pigs and cattle, who consume processed roughage and pasture, which increases the overall land-use burden [62,63]. Nevertheless, the land area and reactive nitrogen emitted (from fertilizers) from the production of chicken feed is significantly higher (more than double) that used to grow plant crop staples (rice, wheat and potatoes) [62]. The total global energy consumption (electricity, natural gas) used in the broiler production system in Europe is estimated to be higher than that for the production of beef or pork [64], although a global data analysis is yet to be undertaken.

What is the potential for broilers to become fossilized? Bird carcasses in the wild are scavenger- and decay-prone and so do not commonly fossilize. Chicken bones, by contrast, are often sold intact within products for human consumption, such as chicken wings, drumsticks and whole birds, and post-consumption the discarded bones form a common component of ordinary landfill sites as part of domestic garbage [65]. The low skeletal density of chicken bones [30] would normally mitigate against long-term preservation potential. However, organic materials are often well preserved within landfill deposits, where anaerobic conditions mean that bones ‘do not so much degrade as mummify’ [66]. The osteo-pathology of modern broiler bones could be used as an additional stratigraphic characteristic of late-twentieth century birds which have been bred for weight gain and/or a fast growth-rate. Further research is needed to document the extent of the occurrence and type of osteo-pathologies in broilers in the twentieth century in order to better constrain their incidence through this time interval.

Carcasses can be disposed of in on-farm burial pits from routine losses during production [67], or as mass-burials at landfills resulting from the depopulation of flocks affected by pathogenic outbreaks of avian influenza, as when 10 million poultry were euthanized in South Korea in 2008 [68]. The broiler chicken is therefore likely to leave a widespread and distinctive biostratigraphic signal in the sedimentary record, as a key fossil index taxon of the Anthropocene. Its potential in this respect is similar to that of other anthropogenic materials which have appeared, or exponentially accumulated in volume. These include during the Great Acceleration [69] from the mid-twentieth century, materials such as plastic, concrete and spheroidal carbonaceous particles [1]. Broilers are globally distributed and their carcasses have accumulated in settings which lead to good fossil preservation potential. Chicken bones, though not as homogeneously distributed as some geochemical anthropogenic markers such as radionuclides [1], will be abundant at landfill sites and other widely distributed accumulations. Given this global distribution, together with its huge population size and distinctive biology, genetics and bone geochemistry, the broiler chicken may be viewed as a key species indicator of the proposed Anthropocene Epoch.

## 5. Conclusion

The advent of the fast-growing broiler morphotype in the 1950s and its uptake across industrial farms worldwide, can be viewed as a near-synchronous global signal of change to the biosphere, currently maintained by humans and the technosphere. Modern broiler chickens are morphologically, genetically and isotopically distinct from domestic chickens prior to the mid-twentieth century. The global range of modern broilers and biomass dominance over all other bird species is a product of human intervention. As such, broiler chickens vividly symbolize the transformation of the biosphere to fit evolving human consumption patterns, and show clear potential to be a biostratigraphic marker species of the Anthropocene.

## Supplementary Material

SI_Figure 1

## Supplementary Material

SI_Figure 2

## Supplementary Material

SI_Figure 3

## Supplementary Material

SI_Figure 4

## Supplementary Material

SI_Table 1

## Data accessibility

All data used in this paper is provided in the electronic supplementary material (four figures and one tables).
