# Supplementary figures and images for: The broiler chicken as a signal of a human reconfigured biosphere
Source: R Soc Open Sci. 2018 Dec 12;5(12):180325. doi: 10.1098/rsos.180325 (PMC6304135; doi:10.1098/rsos.180325)

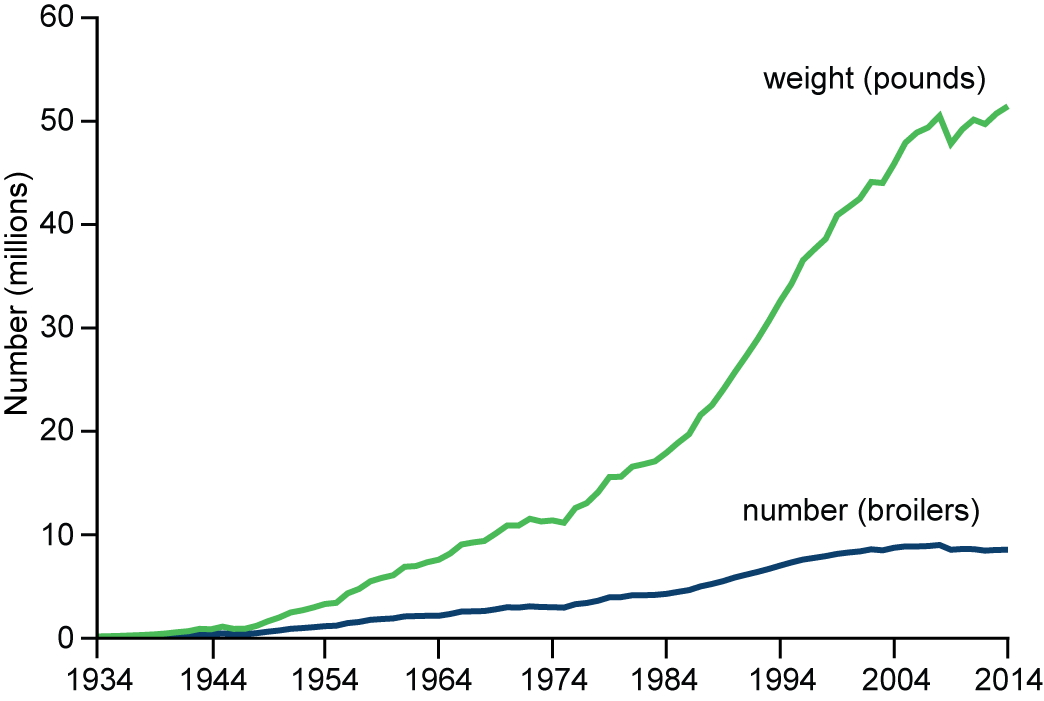

Supplement: SI_Figure 1 [file rsos180325supp1.tif]

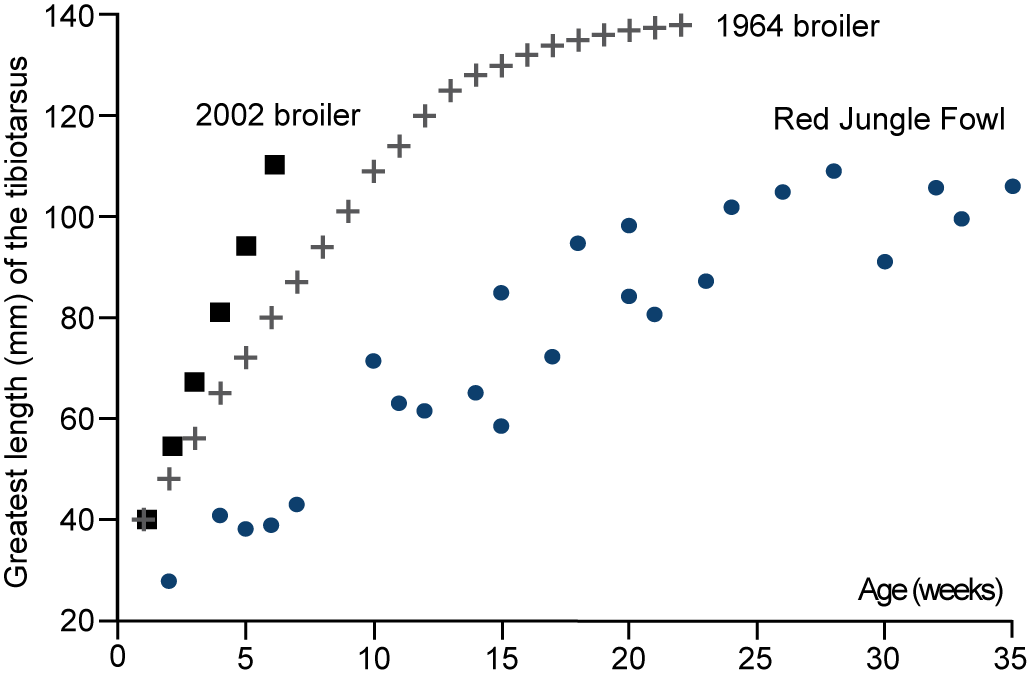

Supplement: SI_Figure 2 [file rsos180325supp2.tif]

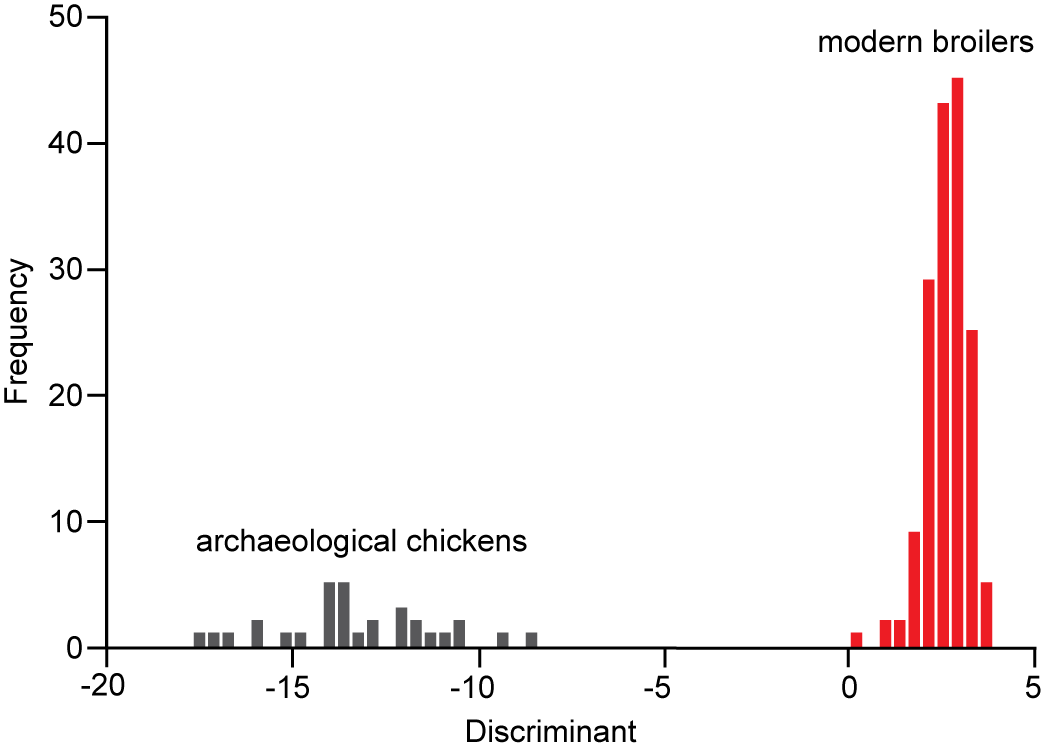

Supplement: SI_Figure 3 [file rsos180325supp3.tif]

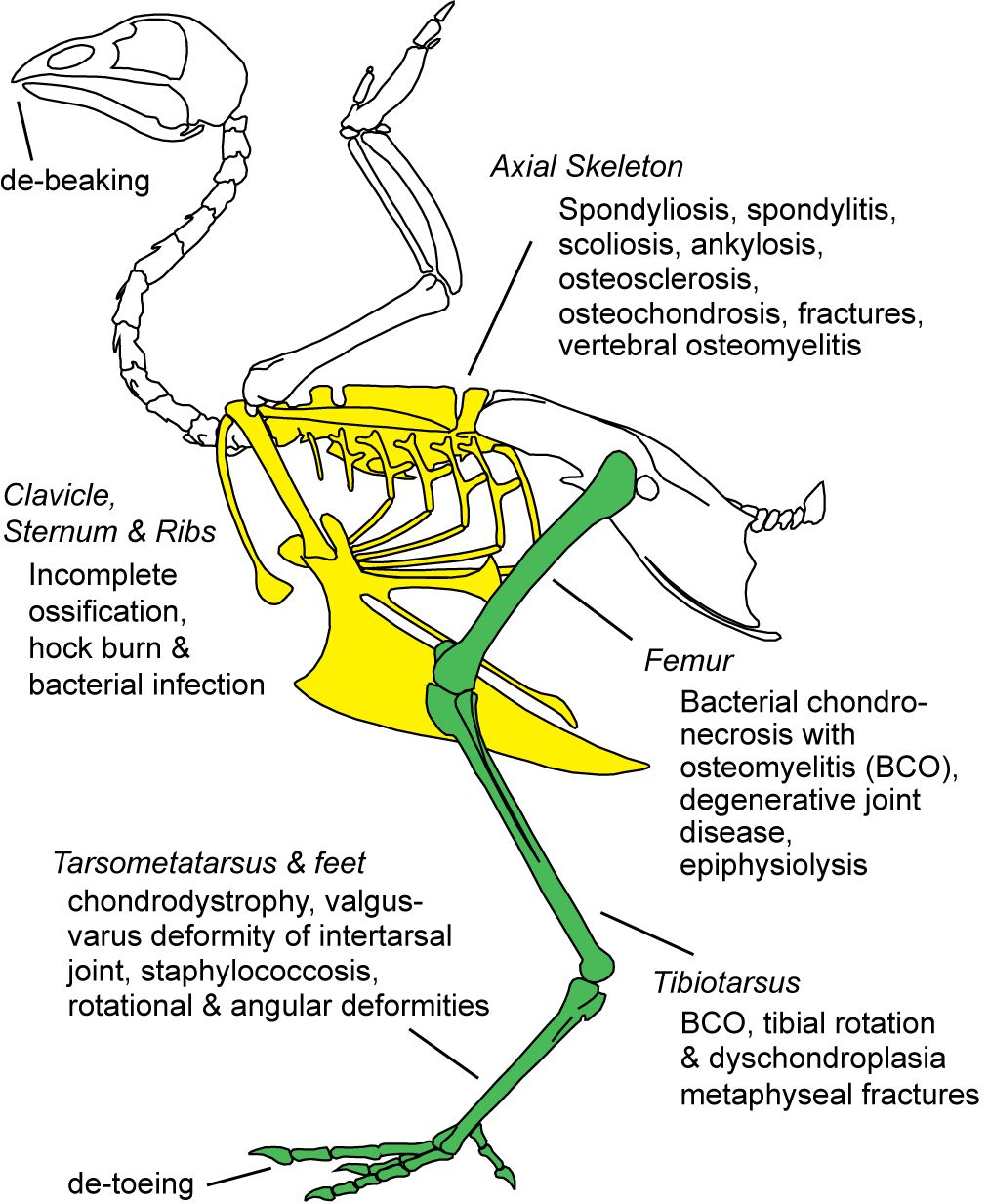

Supplement: SI_Figure 4 [file rsos180325supp4.jpg]
