# Supplementary material for: The broiler chicken as a signal of a human reconfigured biosphere
Source: R Soc Open Sci. 2018 Dec 12;5(12):180325. doi: 10.1098/rsos.180325 (PMC6304135; doi:10.1098/rsos.180325)
Supplement: SI_Table 1 [file rsos180325supp5.docx]

|  | RJF | 40-100CE | 100-300CE | 200-400CE | 650-850CE | 900-1250CE | 1220-1350CE | 1340-1500CE | 1480-1600CE | 1540-1650CE | 1630-1710CE | 1700-1800CE | 1800-1900CE | Ross 308 | Cobb 500 |
| --- | --- | --- | --- | --- | --- | --- | --- | --- | --- | --- | --- | --- | --- | --- | --- |
| N | 6.00 | 29.00 | 35.00 | 24.00 | 44.00 | 126.00 | 59.00 | 44.00 | 50.00 | 25.00 | 23.00 | 12.00 | 15.00 | 16.00 | 14.00 |
| Min | 9.40 | 9.50 | 5.40 | 9.50 | 9.40 | 5.00 | 9.00 | 9.20 | 9.20 | 10.20 | 10.40 | 10.80 | 11.10 | 16.68 | 17.10 |
| Max | 10.90 | 12.40 | 55.00 | 13.50 | 13.20 | 14.40 | 15.60 | 16.00 | 16.10 | 16.80 | 16.60 | 14.20 | 15.50 | 19.88 | 22.10 |
| Mean | 10.37 | 10.70 | 11.77 | 11.28 | 10.80 | 10.67 | 10.79 | 11.67 | 12.42 | 12.93 | 12.59 | 12.02 | 12.39 | 17.90 | 19.55 |
| Std. error | 0.22 | 0.14 | 1.30 | 0.25 | 0.12 | 0.11 | 0.15 | 0.24 | 0.24 | 0.31 | 0.34 | 0.34 | 0.29 | 0.22 | 0.43 |
| Variance | 0.29 | 0.54 | 58.71 | 1.47 | 0.64 | 1.43 | 1.25 | 2.57 | 2.76 | 2.37 | 2.71 | 1.41 | 1.22 | 0.80 | 2.60 |
| Stand. dev | 0.54 | 0.74 | 7.66 | 1.21 | 0.80 | 1.19 | 1.12 | 1.60 | 1.66 | 1.54 | 1.65 | 1.19 | 1.11 | 0.89 | 1.61 |
| Median | 10.50 | 10.50 | 10.50 | 11.10 | 10.70 | 10.50 | 10.40 | 11.25 | 12.00 | 12.20 | 12.30 | 11.80 | 12.00 | 17.83 | 19.56 |
| 25 prcntil | 10.00 | 10.10 | 9.90 | 10.08 | 10.20 | 10.10 | 10.20 | 10.50 | 11.15 | 12.00 | 11.30 | 10.88 | 11.80 | 17.12 | 17.80 |
| 75 prcntil | 10.75 | 11.05 | 11.50 | 12.43 | 11.28 | 11.33 | 11.10 | 12.58 | 13.70 | 14.15 | 14.00 | 12.93 | 13.20 | 18.56 | 20.55 |
| Coeff. var | 5.24 | 6.87 | 65.12 | 10.75 | 7.40 | 11.20 | 10.36 | 13.74 | 13.39 | 11.90 | 13.08 | 9.87 | 8.93 | 5.00 | 8.24 |
|  |  |  |  |  |  |  |  |  |  |  |  |  |  |  |  |
|  |  | 67.5 | 93.5 | 44 | 96.5 | 324 | 162.5 | 60.5 | 26 | 5.5 | 5 | 3 | 0 | 0 | 0 |
| 40-100CE | 0.4047 |  | 481.5 | 258.5 | 601.5 | 1812 | 828 | 393.5 | 242.5 | 55 | 82.5 | 56.5 | 32.5 | 0 | 0 |
| 100-300CE | 0.6847 | 0.7306 |  | 314.5 | 695 | 2125 | 997.5 | 505 | 356.5 | 117 | 138.5 | 85.5 | 80 | 16 | 14 |
| 200-400CE | 0.1522 | 0.1108 | 0.1048 |  | 419 | 1160 | 548 | 458 | 357.5 | 128 | 152 | 96 | 96 | 0 | 0 |
| 650-850CE | 0.2947 | 0.6844 | 0.4616 | 0.1632 |  | 2577 | 1177 | 655.5 | 421 | 94 | 139 | 98.5 | 60.5 | 0 | 0 |
| 900-1250CE | 0.5584 | 0.9469 | 0.7427 | 0.0712 | 0.4873 |  | 3695 | 1786 | 1166 | 302.5 | 421.5 | 276 | 207 | 0 | 0 |
| 1220-1350CE | 0.7503 | 0.8102 | 0.7870 | 0.1081 | 0.4189 | 0.9482 |  | 814 | 526.5 | 173.5 | 191.5 | 117.5 | 114 | 0 | 0 |
| 1340-1500CE | 0.0338 | 0.0059 | 0.0090 | 0.3719 | 0.0091 | 0.0004 | 0.0013 |  | 809 | 288.5 | 333 | 215.5 | 206 | 0 | 0 |
| 1480-1600CE | 0.0011 | 0.0000 | 0.0000 | 0.0052 | 0.0000 | 0.0000 | 0.0000 | 0.0276 |  | 484 | 537 | 259.5 | 341.5 | 0 | 0 |
| 1540-1650CE | 0.0005 | 0.0000 | 0.0000 | 0.0006 | 0.0000 | 0.0000 | 0.0000 | 0.0011 | 0.1141 |  | 248.5 | 93.5 | 135 | 1 | 0 |
| 1630-1710CE | 0.0006 | 0.0000 | 0.0000 | 0.0085 | 0.0000 | 0.0000 | 0.0000 | 0.0226 | 0.6558 | 0.4262 |  | 109.5 | 170.5 | 0 | 0 |
| 1700-1800CE | 0.0023 | 0.0008 | 0.0025 | 0.1101 | 0.0010 | 0.0003 | 0.0003 | 0.3372 | 0.4757 | 0.0686 | 0.3294 |  | 69.5 | 0 | 0 |
| 1800-1900CE | 0.0005 | 0.0000 | 0.0001 | 0.0157 | 0.0000 | 0.0000 | 0.0000 | 0.0314 | 0.6070 | 0.1451 | 0.9642 | 0.3279 |  | 0 | 0 |
| Ross 308 | 0.0005 | 0.0000 | 0.0000 | 0.0000 | 0.0000 | 0.0000 | 0.0000 | 0.0000 | 0.0000 | 0.0000 | 0.0000 | 0.0000 | 0.0000 |  | 44 |
| Cobb 500 | 0.0006 | 0.0000 | 0.0000 | 0.0000 | 0.0000 | 0.0000 | 0.0000 | 0.0000 | 0.0000 | 0.0000 | 0.0000 | 0.0000 | 0.0000 | 0.0050 |  |

Table 1. Statistical analysis of tibiotarsus distal breadth measurements shown in Figure 3. Measurements are in mm. Univariate statistics are presented in the upper register of the table. The test (U) statistics (top right of the lower register) and p-values (bottom left of the lower register) of a non-parametric Mann-Whitney pairwise comparison are presented. Mann-Whitney analysis is commonly used in zooarchaeological studies to test temporal changes to animal bone measurements^77, 78^. Statistically-significant p-values are highlighted in grey. The null hypothesis, that there is no difference in median tibiotarsus distal breadth measurements over time, can be rejected for chicken bones younger than 1340-1450C.E. Modern broiler tibiotarsi are significantly larger than those from all other periods.
